# The Factor Structure of the Forms of Self-Criticising/Attacking & Self-Reassuring Scale in Thirteen Distinct Populations

**DOI:** 10.1007/s10862-018-9686-2

**Published:** 2018-06-13

**Authors:** Júlia Halamová, Martin Kanovský, Paul Gilbert, Nicholas A. Troop, David C. Zuroff, Nicola Hermanto, Nicola Petrocchi, Marion Sommers-Spijkerman, James N. Kirby, Ben Shahar, Tobias Krieger, Marcela Matos, Kenichi Asano, FuYa Yu, Jaskaran Basran, Nuriye Kupeli

**Affiliations:** 1 Institute of Applied Psychology, Faculty of Social and Economic Sciences, Comenius University in Bratislava, Mlynské luhy 4, 821 05 Bratislava, Slovakia; 2 Institute of Social Anthropology, Faculty of Social and Economic Sciences, Comenius University in Bratislava, Bratislava, Slovakia; 3 Centre for Compassion Research and Training, College of Health and Social Care Research Centre, School of Sciences, University of Derby, Derby, UK; 4 Department of Psychology and Sports Sciences, School of Life and Medical Sciences, University of Hertfordshire, Hertfordshire, Hatfield UK; 5 Department of Psychology, McGill University, Quebec, Montréal Canada; 6 Department of Economics and Social Sciences, John Cabot University, Rome, Italy; 7 Centre for eHealth and Wellbeing Research, University of Twente, Enschede, The Netherlands; 8 The School of Psychology, The University of Queensland, Brisbane, Australia; 9 Paul Baerwald School of Social Work and Social Welfare, Hebrew University of Jerusalem, Jerusalem, Israel; 10 Clinical Psychology and Psychotherapy, University of Bern, Bern, Switzerland; 11 Cognitive and Behavioural Centre for Research and Intervention, University of Coimbra, Coimbra, Portugal; 12 Research Center for Child Mental Development, Chiba University, Chiba, Japan; 13 Student counseling center K-12 Education Administration, Ministry of Education, Yilan City, Taiwan; 14 The Compassionate Mind Foundation, Derby, UK; 15 Marie Curie Palliative Care Research Department, University College London, TottenhamCourt Road 149, London, W1T 7NF UK

**Keywords:** Self-criticism, Self-reassurance, Bifactor models, Two-tier model, Cross-cultural studies

## Abstract

There is considerable evidence that self-criticism plays a major role in the vulnerability to and recovery from psychopathology. Methods to measure this process, and its change over time, are therefore important for research in psychopathology and well-being. This study examined the factor structure of a widely used measure, the Forms of Self-Criticising/Attacking & Self-Reassuring Scale in thirteen nonclinical samples (*N* = 7510) from twelve different countries: Australia (*N* = 319), Canada (*N* = 383), Switzerland (*N* = 230), Israel (*N* = 476), Italy (*N* = 389), Japan (*N* = 264), the Netherlands (*N* = 360), Portugal (*N* = 764), Slovakia (*N* = 1326), Taiwan (*N* = 417), the United Kingdom 1 (*N* = 1570), the United Kingdom 2 (*N* = 883), and USA (*N* = 331). This study used more advanced analyses than prior reports: a bifactor item-response theory model, a two-tier item-response theory model, and a non-parametric item-response theory (Mokken) scale analysis. Although the original three-factor solution for the FSCRS (distinguishing between Inadequate-Self, Hated-Self, and Reassured-Self) had an acceptable fit, two-tier models, with two general factors (Self-criticism and Self-reassurance) demonstrated the best fit across all samples. This study provides preliminary evidence suggesting that this two-factor structure can be used in a range of nonclinical contexts across countries and cultures. Inadequate-Self and Hated-Self might not by distinct factors in nonclinical samples. Future work may benefit from distinguishing between self-correction versus shame-based self-criticism.

## Introduction

There is considerable evidence that when confronted with life difficulties and setbacks, the way we make attributions of causality for those setbacks (e.g. self-blame vs. external blame) and the way we evaluate, judge, condemn or accept, and support ourselves has a major impact on our coping, resilience, recovery, and perseverance (Blatt 2004; Ehret et al. 2015; Gilbert and Irons 2005; Mandel et al. 2015; Shahar 2015; Zuroff et al. 2004; Zuroff et al. 2005). There is extensive literature base showing that self-criticism, which involves negative self-labelling and harsh judgement (Kannan and Levitt 2013; Shahar 2015), along with negative emotions such as anger and contempt with the self (Whelton and Greenberg 2005), is associated with vulnerabilities to various forms of psychopathology (Shahar 2015; Zuroff et al. 2005). It is therefore a commonly target of psychotherapeutic intervention (Gilbert and Irons 2005; Kannan and Levitt 2013; Kirby and Gilbert 2017; Leaviss and Uttley 2015; Shahar 2014; Shahar et al. 2012).

Self-criticism, however, can be defined and measured in different ways. For example, one of the first measures of self-criticism was the Depressive Experiences Questionnaire, which assesses self-criticism, dependency, and self-efficacy (DEQ; Blatt et al. 1976). The DEQ is a well-validated scale and has been extensively used by clinical researchers to demonstrate significant links between self-criticism and psychopathology, particularly depression (see Zuroff et al. 2005 for a review). Related to this measure is the Levels of Self-Criticism Scale (LOSC; Thompson and Zuroff 2004). This scale measures both comparative self-criticism (e.g., “I fear that if people get to know me too well, they will not respect me.”), and internalized self-criticism (e.g., “Failure is a very painful experience for me.”). The former refers to negative thoughts and feelings experienced when comparing oneself to others, whilst the latter reflects self-critical thoughts and feelings resulting from failure to meet personal standards or goals. A more recent assessment of self-criticism is represented by The Self-Critical Rumination Scale (e.g., “My attention is often focused on aspects of myself that I’m ashamed of.”; “I always seem to be rehashing in my mind stupid things that I’ve said or done.”) (Smart et al. 2016). All three scales are highly correlated and strongly correlated with depression (Smart et al. 2016). The only scale assessing situational state self-criticism is The Self-Compassion and Self-Criticism Scales (SCCS; Falconer et al. 2015). It consists of five imaginary scenarios (e.g. “*You arrive home to find that you have left your keys at work*.”), to which responses indicate varying degrees of situational self-criticism (Contemptuous reaction) or situational self-compassion (Soothing reaction).

Building on earlier research (Blatt et al. 1976), and suggestions by Driscoll (1989) that self-criticism can take different forms and serve different functions, Gilbert (1989, 2016) took an evolutionary functional analysis perspective on self-evaluations, specifically in relation to self-criticism and self-reassurance. Consequently, Gilbert et al. (2004) developed measures that sought to distinguish different forms and functions of self-criticism. For example, some individuals criticise themselves in the belief that it will help improve and motivate them to achieve, essentially assigning self-criticism a positive function. Conversely, others can be critical because they dislike or want to get rid of different parts of the self rather than improve them. Thus, the specific self-critical function influence how people feel, behave, and think in relation to themselves. In addition, based on evolutionary models, it was suggested that a focus on inadequacy or sense of inferiority is linked to social comparison processes and fitting within a group; that is, one feels inadequate in relation to a desired social standard (Gilbert et al 2004). Self-hating, on the other hand, relies on a different type of process that evolved for differentiating oneself from out-groups. When self-hating is directed to parts of the self and compared with self-inadequacy, it is more closely linked to emotions such as disgust and wanting to be rid of and even destroy parts of the self. In essence, one relates to parts of oneself as though these parts were an out-group. It is also suggested that self-hatred is more pathogenic than is self-inadequacy (Gilbert et al. 2004).

Based on this evolutionary model of self-criticism, two scales were developed by Gilbert et al. (2004), the Forms of Self-criticising/Attacking and Self-reassuring scale (FSCRS) and the Functions of Self-Criticizing/Attacking Scale (FSCS). It was hypothesised that two distinct forms of self-criticism could be identified, one linked to the sense of inadequacy and wanting to improve, and the other linked to self-dislike and even self-hatred, and wanting to remove or get rid of undesired aspects of the self. Moreover, it was hypothesised that these forms of self-criticism are linked to different degrees of psychopathology (Gilbert 2016). Preliminary evidence supported the ability of the scale to distinguish between these two factors of self-criticism, and that indeed self-hating is more strongly linked to psychopathology than a sense of inadequacy (Baião et al. 2015; Gilbert et al. 2004, 2017).

In contrast to self-criticism, self-reassurance is defined as the ability to be self-validating, supportive, compassionate, and bring to mind positive qualities of the self when confronting setbacks. Self-reassurance is associated with improved coping abilities, resilience, and perseverance (Gilbert et al. 2004; Hermanto and Zuroff 2016; Hermanto et al. 2016; Kirby 2016). Self-reassuring or compassionate orientations to oneself and others are associated with a range of beneficial physiological processes and psychological well-being outcomes (Keltner et al. 2014). For example, there is increasing evidence that supportive, validating, and compassionate approaches to the self lead to benefits through different neurophysiological systems compared to self-criticism (Longe et al. 2010). Compassion training may impact physiological indicators of well-being and even impact processes such as telomere length – bits of chromosomes that are a biological indicator of aging (Fredrickson et al. 2013). Hence, helping clients to develop capacity to be self-validating, supportive, and compassionate in the face of setbacks is an important therapeutic endeavour given increasing evidence of the effectiveness of compassion-based interventions (Gilbert 2010; Kirby 2016; Kirby et al. 2017b; Leaviss and Uttley 2015; Shahar et al. 2012). Accordingly, the self-reassurance subscale was developed to explore people’s abilities to remember positive qualities about themselves, to provide themselves encouragement when things go wrong, and despite making mistakes to be able to still like themselves (Gilbert et al. 2004). Measuring self-reassurance is crucial both for examining how lack of reassurance is associated with different psychological difficulties and for assessing the effectiveness of interventions designed to enhance this process.

There is increasing evidence that self-criticism and self-reassurance are not simply mirror images of each other. That is, they are not bipolar constructs and therefore should not be combined into a single measure. For example, it is now well established that psychopahology and mental health are not on a single continuum (Lamers et al. 2015; Westerhof and Keyes 2010). Similarly, positive and negative affects are not bipolar but orthogonal constructs and should be measured separately (Mineka et al. 1998; Watson et al. 2008). Mental health and even well-being are not simply due to the absence of self-criticism, but depend on more specific prosocial, validating, and supportive orientations to the self. There is emerging evidence that self-criticism is associated with threat affect and vulnerabilities to mental health problems, whereas compassionate self-validation is associated with affiliative affect and supports the development of well-being. The interaction between these processes is complex (Gilbert et al. 2017; Lamers et al. 2015). Moreover, as we understand more about the physiological processes underlying compassion and compassion training and its impact on the brain (Vrtička et al. 2017) and body (Stellar and Keltner 2017), the more we begin to realise how compassion can stimulate different physiological processes than threat-based criticism (Keltner et al. 2014; Longe et al. 2010). Therefore, self-criticism and self-reassurance are to be regarded as two distinct processes, and therefore should not combined to reflect a single factor based on physiological, psychological, clinical or statistical grounds.

### The origins and development of the FSCRS scale

The FSCRS was developed by Gilbert et al. 2004 on the basis of clinical work with depressed patients who expressed a variety of thoughts related to self-criticism and self-reassurance. In its original form, the scale comprises three subscales: Inadequate-Self (IS), which focuses on feelings of personal inadequacy, Hated-Self (HS) measuring the desire to hurt or punish oneself, and Reassured-Self (RS) which is an ability to reassure and support the self. To date, the English version of the FSCRS has been translated into ten other languages including Chinese (Yu, personal communication), Dutch (Sommers-Spijkerman et al. 2017), French (Gheysen et al. 2015), German (Wiencke, personal communication), Hebrew (Shahar et al. 2015), Italian (Petrocchi and Couyoumdjian 2016), Japanese (Kenichi, personal communication), Portuguese (Castilho et al. 2015), Slovak (Halamová et al. 2017), and Swedish (Lekberg and Wester 2012).

### Psychometric properties of the FSCRS scale

### Reliability of the FSCRS

Internal consistency for the subscales of the FSCRS is generally high. In the development study, Cronbach’s alphas were 0.90, 0.86, and 0.86 for the IS, HS and RS subscales, respectively (Gilbert et al. 2004). Similarly, another large UK study found alphas of 0.91, 0.86, and 0.88, respectively for these subscales (Kupeli et al. 2013). A sample collated from 12 studies reported alphas of 0.90, 0.85, and 0.85 for nonclinical participants, and 0.91, 0.87, and 0.85 for clinical participants, respectively (Baião et al. 2015). In a Portuguese sample, Castilho et al. (2015) demonstrated the test–retest reliability of the FSCRS by administering it twice to 41 participants over a four-week interval. Pearson’s correlation coefficients for the subscales demonstrated sufficient test-retest reliability: IS = 0.72, HS = 0.78, and RS = 0.65. Collectively, these studies demonstrate that the FSCRS has high internal consistency and adequate test-retest reliability.

### Validity of the FSCRS

In the original study (Gilbert et al. 2004), construct validity was examined by comparing the FSCRS with the LOSC (Thompson and Zuroff 2004) scale, which also measures self-criticism. Similar to the FSCRS, the LOSC measures self-criticism as a multi-dimensional construct that takes various forms: comparative and internalised self-criticism. Correlational analyses demonstrated a significant relationship between the FSCRS and LOSC. Pearson correlations between the LOSC Internalised self-criticism subscale and the subscales of the FSCRS were: IS (*r* = 0.77), HS (*r* = 0.57), and RS (*r* = −0.45). Similarly, strong relationships between the LOSC Comparative self-criticism and IS (*r* = 0.63), HS (*r* = 0.55), and RS *(r* = −0.63) were reported.

Castilho et al. (2015) also provided evidence for the construct validity of the FSCRS by comparing the FSCRS subscales with the Self-Compassion Scale (SCS; Neff 2003) subscales. A strong correlation between the SCS and the IS (*r* = −0.63), HS (*r* = −0.53), and RS (*r* = 0.56) was reported. Halamová et al. (2017) examined convergent and divergent validity of the FSCRS by assessing the relationship between the FSCRS and other related instruments and their respective dimensions, specifically the LOSC (Thompson and Zuroff 2004), the SCS (Neff 2003), and the Self-Compassion and Self-Criticism Scale (SCCS; Falconer et al. 2015). In addition, Halamová and Kanovský (2017) also examined the relationship between the FSCRS and the Self-criticism subscale of the Depressive Experiences Questionnaire (DEQ; Blatt et al. 1976). Correlations were in line with the theoretical expectations, indicating that all subscales of the FSCRS have good convergent and divergent validity.

### Factor structure of the FSCRS

According to Gilbert et al. (2004) the scale has a three-factor solution. That IS and HS subscales are separable factors is also supported by evidence of differential associations with other variables. For example, while women score higher on the IS subscale than men (and lower on RS), there is no significant gender difference on the HS subscale (Kupeli et al. 2013). Furthermore, HS is a unique predictor of self-inflicted harm, depression, anxiety, and stress (Gilbert et al. 2004;Gilbert 2010; Kupeli et al. 2017; Xavier et al. 2016), while IS is uniquely associated with the use of self-criticism for self-correction rather than self-punishment (Gilbert et al. 2004). Research in clinical samples also shows that there is a floor effect in HS while there is a full distribution range of scores in the IS scale (Longe et al. 2010).

Kupeli et al. (2013), used confirmatory factor analysis (CFA) to confirm a three-factor solution of the scale. This study reported a strong correlation between the IS and HS subscales, thus suggesting that these subscales reflect a global assessment of self-criticism. However, Kupeli et al. (2013) still concluded that the three-factor model is the most appropriate statistical solution when compared to the single factor and two-factor models. Although the factor structure reported in this study was similar to the original 22-item measure (Gilbert et al. 2004), the authors applied several modifications which resulted in a shortened, 18-item version of the FSCRS. These modifications did not have a detrimental effect on the psychometric quality of the FSCRS scale, but resulted in a reduction in the correlation between the IS and HS subscales. However, all other studies continue to use the original 22-item version.

In a Portuguese sample, Castilho et al. (2015) confirmed a three-factor model in both clinical and nonclinical samples. In nonclinical samples, fit of all confirmatory models (including three-factor model) was suboptimal, but this is possibly the effect of the Maximum Likelihood (ML) estimator, which is not recommended for categorical (ordinal) data. The authors declared that they inspected normality and presence of outliers, but they did not report the results. It can be suggested that multivariate normality cannot be assumed in this case, so the ML estimator distorted the fit indices and most likely the estimation of parameters (Li 2016).

Baião et al. (2015) combined data from 12 previous studies, each with separate samples, and used CFA to test the factor structure of the FSCRS for both nonclinical and clinical samples. The results showed good fit with the data for the three-factor model of the FSCRS (Baião et al. 2015) measuring the two forms of self-criticism (IS and HS) and self-reassurance (RE). However, Baião et al. (2015) used a ML estimator too, which is biased for ordinal data displaying the multivariate non-normality (Finney and DiStefano 2008). Although authors report skewness and kurtosis for items, they do not report results for the multivariate non-normality (Mardia’s test) available in AMOS. They also tested the two-factor model (IS and HS merged) to demonstrate that its poor fit raises doubts about two dimensions of self-criticism and self-reassurance.

In contrast, several studies have also reported that the correlation between the two factors of self-criticism (IS & HS) range between 0.68 and 0.73 (Gilbert et al. 2004; Irons et al. 2006; Kupeli et al. 2013), with one study reporting the correlation as high as 0.81 (Halamová et al. 2017). High inter-correlations between the IS and HS subscales suggest a risk of multicollinearity and caution must be taken when developing predictive models (Howell 2002). Recently, researchers have postulated as to whether IS and HS can be merged into one factor which reflects a global measure of self-criticism, and suggest that the FSCRS consists of two factors, self-criticism (IS + HS) and self-reassurance (RS) (Gilbert et al. 2006a, b; Halamová et al. 2017; Richter et al. 2009; Rockliff et al. 2011).

In a recent study, Halamová et al. (2017) used Item Response Theory (IRT) and robust linear confirmatory factor analyses to confirm the three-dimensional structure of the FSCRS, and unlike previous studies, a two-dimensional structure (in which IS and HS are merged) also proved a good fit with data.

Furthermore, there may be some statistical limitations of previous studies - they did not use a polychoric matrix and logistic estimation to take account of the ordinal nature of the items, nor corrections for non-normal distributions (Li 2016; Finney and DiStefano 2008). Kupeli et al. (2013) and Castilho et al. (2015) used linear methods of the confirmatory factor analysis. Kupeli et al. (2013) used a WLSMV estimator, which is more appropriate for categorical data than the ML estimator used by Castilho et al. (2015) and Baião et al. (2015). For ordinal multivariate analysis, logistic methods, namely IRT, are more optimal (Maydeu-Olivares et al. 2011; Kankaraš et al. 2011).

To conclude, debate concerning the factor structure of the FSCRS still remains open; thus, the examination of the issue in several samples will be useful in providing a more comprehensive understanding of the FSCRS factor structure.

### Aim of the current study

To summarise, no study to date has examined the psychometric properties and factor structure of the FSCRS across multiple language versions using advanced statistical methods such as bifactor and two-tier models. Building on previous research on the FSCRS, the aim of this study was to examine the factor structure of the FSCRS across thirteen different populations and eight language versions. In addition to two-factor and three-factor models used in previous studies, bifactor models and two-tier models were computed to examine whether: 1) the original three-factor model consisting of HS, IS, and RS is confirmed; 2) the use of a single overall FSCRS score, as suggested by some practitioners, is justified psychometrically; and 3) the use of two dimensions of Self-Reassurance (RS) and Self-Criticism (HS and IS) is supported.

## Methods

### Measuring instrument

#### The Forms of Self-criticising/Attacking & Self-Reassuring Scale

(FSCRS; Gilbert et al. 2004) is a 22-item instrument, which was developed to determine the level of self-criticism and the ability to self-reassure when one faces setbacks and failure. Participants use a 5-point Likert scale to rate the extent to which various statements are true about them (1 = not at all like me; 5 = extremely like me). The first of the three factors, IS, is comprised of nine items that capture the experiences of failure, setback, inadequacy, and defeat, for example: “I think I deserve my self-criticism.”, “I remember and dwell on my failings.”, and “I am easily disappointed with myself.”. The second factor, HS, consists of five items. It captures a destructive disposition to the self, characterized by hatred, contempt, disgust, aggression, and even sadistic desires to harm or attack oneself. Items that load on this factor include: “I have become so angry with myself that I want to hurt or injure myself.” or “I feel a sense of disgust with myself.” (Gilbert et al. 2004). The third factor, RS, consists of seven items, and captures the capacity to be self-soothing and consider the self with encouragement, support, and validation when faced with negative events. It focuses on positive memories and past successes and results in confidence and tolerance during vulnerability. Items that represent this factor include “I still like being me.”, “I am able to remind myself of positive things about myself.” and “I encourage myself for the future.”.

### Sampling procedure

Various samples using the FSCRS were collected by emailing the authors of published research studies and research projects. We identified articles by searching using Google Scholar using search terms such as “the forms of self-criticising/attacking & self-reassuring scale” or “FSCRS”. The first author of this article then emailed all corresponding authors of studies with at least 215 nonclinical participants in a sample, which is a minimum sample size to perform the required statistical methods (Velicer and Fava 1998). In addition, we referred to the Compassionate Mind website (https://compassionatemind.co.uk/uploads/files/research-register-for-website.pdf) to locate and contact authors of yet unpublished research projects. Altogether, the first author of this study sent approximately 40 emails with requests for cooperation. Out of those, 13 researchers agreed to provide their FSCRS data. Therefore, the current analysis includes data of 13 different non-clinical samples.

### The samples and procedures from different countries

Out of 11 existing language versions of FSCRS currently available, this study includes data from eight. The complete data set consists of 5 distinct English language samples from 4 different countries including Australia (*N* = 319), Canada (*N* = 383), the United Kingdom 1 (*N* = 1570), the United Kingdom 2 (*N* = 883), and USA (*N* = 331). There were also samples from seven other language translations namely Chinese (*N* = 417), Dutch (*N* = 360), German (*N* = 230), Hebrew (*N* = 476), Italian (*N* = 389), Japanese (*N* = 264), Portuguese (*N* = 764), and Slovak (*N* = 1.326). In total, we tested 13 distinct nonclinical samples with an overall sample size of 7510. In all these samples, data were collected in accordance with the ethical standards of the institutional and/or national research committee and with the 1964 Helsinki declaration and its later amendments or comparable ethical standards.

### Chinese version of the FSCRS from Taiwan

Participants from Taiwan were recruited from universities by online survey, through social media and also by paper tests between students (Yu 2013). A sample of 417 participants took part of whom 56.1% were female (*N* = 234), 41.7% were male (*N* = 174), and 2.2% did not provide this information (*N* = 9). The mean age was 22.7 years (*SD* = 4.27), and ranged from 18 to 58 years. The Chinese version of the FSCRS was back translated in order to check its accuracy.

### Dutch version of the FSCRS from the Netherlands

A total number of 360 participants, ranging from 18 to 81, participated (Sommers-Spijkerman et al. 2017) of which 64.4% were female (*N* = 232) and 35.6% were male (*N* = 128). The mean age was 30.8 years (*SD* = 13.4). A convenience sample of participants was recruited by various students to an online cross-sectional survey conducted at a university. The Dutch version of the FSCRS was back translated in order to check its accuracy.

### English version of the FSCRS from Australia

The participants were Australians selected from a larger sample of participants from the general population (Kirby et al. 2017a). Convenience sampling was used to recruit participants to an online survey. The research sample from Australia consisted 319 participants of whom 47 were males (14.7%) and 272 females (85.3%). The mean age was 41.3 years (*SD* = 14.2), and ranged from 17 to 87 years.

### English version of the FSCRS from Canada

Participants were 381 undergraduate and graduate students at a large Canadian university (143 men [37.5%], 238 women [62.5%]), ranging in age from 18 to 49 years old (*M* = 21.1, *SD* = 3.4). The samples (Hermanto and Zuroff 2016, 2017; Zuroff et al. 2016) were recruited online through various university advertisements and the university pool of psychology research participants.

### English version of the FSCRS from the United Kingdom 1

Participants from the first UK sample were recruited from a university and through social networking sites and health and well-being forums (Kupeli et al. 2013) to an online survey. For the overall sample of 1570, mean age was 28.5 (*SD* 10.7) with range from 18 to 71, 1295 participants were female (82.5%) and 275 were male (17.5%).

### English version of the FSCRS from the United Kingdom 2

The second UK sample were students recruited from a university. Participants completed pen and paper questionnaires. There were 883 participants of whom 672 were women (76.1%) and 210 were men (23.8%). The mean age was 24.1 (*SD* = 7.8) with a range between 18 and 57. The dataset comprised of data collected from various research studies (Baião et al 2015; Gilbert et al. 2006a, b; Gilbert and Miles 2000; Gilbert et al. 2002, 2004, 2005, 2012).

### English version of the FSCRS from USA

The USA population was obtained from a university (Gilbert et al. 2017). Participants were recruited via online participant management software. The final sample included 331 participants of whom 89 were males (26.9%) and 242 females (73.1%). The mean age was 20.8 years (*SD* = 5.3), and ranged between 18 to 58 years.

### German version of the FSCRS from Switzerland

Participants were recruited in the German-speaking part of Switzerland through a study website and postings on internet forums. Participants were directed to an online survey from search engines or links from other websites (Krieger et al. 2016; Krieger, personal communication). The Swiss sample included 230 participants, of whom 66 were males (29%) and 164 females (71%). The mean age was 38.9 years (*SD* = 14.3), and ranged from 19 to 76 years. The German version of the FSCRS was back translated (Wiencke, personal communication).

### Hebrew version of the FSCRS from Israel

The Israeli sample consisted of 476 participants (199 males [41.9%] and 276 females [58.1%]) from the general population who were recruited via an online survey platform and undergraduate students from a private college (Shahar et al. 2015; Shahar, personal communication). The mean age was 30.6 years (*SD* = 11.8), and ranged from 18 to 64 years. The Hebrew version of the FSCRS was not back translated.

### Italian version of the FSCRS from Italy

This study (Petrocchi and Couyoumdjian 2016) was conducted through an online survey and participants were recruited via both an Italian university students mailing list, and other professional mailing lists and web advertising. The research sample from Italy included 393 participants of whom 111 were males (28.5%) and 278 females (71.5%). The mean age was 33.2 years (*SD* = 10.8), and ranged from 18 to 76 years. The Italian version of the FSCRS was back translated.

### Japanese version of the FSCRS from Japan

The research sample from Japan included 264 participants of whom 47 were males (17.8%) and 214 females (81.1%) (Kenichi, personal communication). The mean age was 18.8 years (*SD* = 1.1), and ranged from 18 to 28 years. Participants were students attending a course in psychology at university. The Japanese version of the FSCRS was not back translated.

### Portuguese version of the FSCRS from Portugal

The research sample from Portugal included 764 participants of whom 162 were males (21.2%) and 600 females (78.5%) (Gilbert et al. 2017). The mean age was 27.9 years (*SD* = 11.2), and ranged from 16 to 65 years. Convenience sampling was used to recruit participants using an online platform from a university setting and from the general population. The Portuguese version of the FSCRS was back translated.

### Slovak version of the FSCRS from Slovakia

Data were obtained by convenience sampling; questionnaires were distributed in paper format and as an online survey via social networks (Halamová et al. 2017). The research sample from Slovakia included 1326 participants of whom 422 were males (31.8%) and 904 females (68.2%). The mean age was 29.6 years (*SD* = 12.1), and ranged from 17 to 82 years. The Slovak version of the FSCRS was back translated.

### Data analysis

For data management, we used the software SPSS Statistics-20, and for the statistical processing, program R (Version 3. 1. 3, R Core Team 2015), the library mirt (Chalmers 2012), and mokken (Van der Ark 2012).

**Fig. 1:**
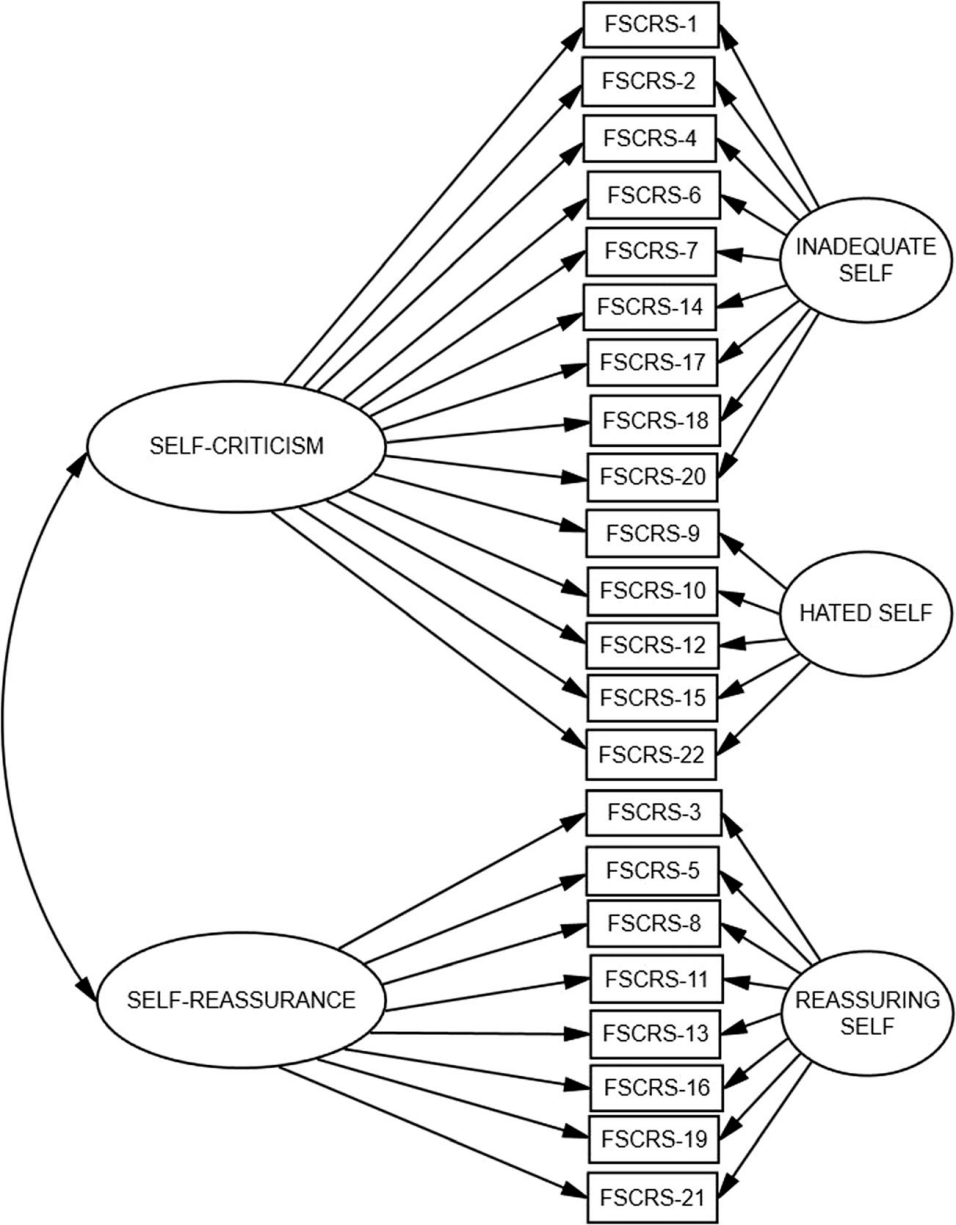
Two-tier model of the FSCRS scale. *Note*. FSCRS1-FSCRS22 particular items of FSCRS with numbers

We checked the fit of several models: the two-factor correlated model (where IS and HS dimensions are merged), the three-factor correlated model, the bifactor model, and the two-tier model (two primary dimensions: the Self-criticism consisting of items from the IS and HS subscales, and Self-Reassurance – see Fig. 1).

For a better understanding of the various results of the FSCRS factor analyses and their interpretation, we now briefly describe the essential aspects of the different factorial models below, as well as their respective interpretations. The three-factor correlated model has only two sources to capture variance in items: latent factor(s) on the one hand, and error on the other. It does not allow the partition of variance among general factor(s), domain specific group factors, and error. If there is domain specific content in items after the extraction of general factor(s), this model does not account for it. The bifactor model (Reise et al. 2013) allows the separation of general and specific factors, so the contribution of the three specific factors can be studied independently of the general factor: in other words; we can inspect how much variance is explained by the single general factor (Self-criticism) in comparison to variance explained by the three specific factors (IS, HS, and RS) controlling for the global factor of self-criticism. Conceptually, it is variance explained by the inadequacy, reassurance, and hate factors after extracting global self-criticism. The extent of such variance is assessed with the hierarchical ω in Table 2; for example, the value of the hierarchical ω = 0.81 means that 81% of variance is explained by the single general factor – Self-criticism – and 19% of variance is explained by three specific factors and error. The explained common variance (ECV) in Table 2 decomposes the explained variance between the general factor and specific factors; for example, the value of the ECV 0.90 means that 90% of explained variance is accounted for by the general factor, and 10% of explained variance is accounted for by specific factors. The two-tier model (Bonifay 2015; Cai 2016) shares this decomposition of the explained variance, with the only difference being that it has two general factors (Self-criticism and Self-reassurance) instead of one. By direct comparison of the two-tier model and the bifactor model, we can see whether there are two general sources of explained variance over and above the contributions of the specific factors, or a single common source of explained variance over and above the contributions of the specific factors. The following six-stage process was undertaken to evaluate the factor structure and psychometric properties of the FSCRS:

1. For each sample, we fitted the IRT confirmatory two-factor correlated model (where IS and HS dimensions are merged), and the three-factor correlated model. We assume that IRT models are more accurate if data are ordinal due to the logistic nature of their estimation (Maydeu-Olivares et al. 2011). We used GRM (graded response model) estimation (Gibbons et al. 2007), and the Metropolis-Hastings Robbin-Munro algorithm. We reported standard fit indices: Comparative Fit Index (CFI), Tuker-Lewis Index (TLI), Root Mean Square Error of Approximation (RMSEA), Standardised Root Mean square Residual (SRMR), Akaike Information Criterion (AIC), and Bayesian Information Criterion (BIC). Each three-factor model was compared with the two-factor model by means of the likelihood ratio test. For confirmatory IRT models, the standard cutoff criteria for fit indices were used: CFI and TLI ˃ 0.90 indicate acceptable fit, CFI and TLI ˃ 0.95 indicate excellent fit; RMSEA (and SRMR) ˂ 0.08 indicate acceptable fit, and ˂ 0.05 indicate excellent fit (Hu and Bentler 1999). The model with the lowest BIC is preferred (Raftery 1995). We considered a model to show acceptable fit if, and only if, all four indices were at least acceptable in order to prevent selection bias.
2. We fitted the IRT confirmatory bifactor models with 22 items (one general factor, three specific factors), for each sample. We used Graded Response Model (GRM) estimation (Gibbons et al. 2007). Again, we reported the following standard fit indices: CFI, TLI, RMSEA, SRMR, and information criteria AIC and BIC. Each bifactor model was compared with the three-factor model by means of the likelihood ratio test.
3. After evaluating the fit of those models, we computed four measures of reliability for each model using all 22 items of the FSCRS: Cronbach’s alpha, Omega, Hierarchical Omega, and ECV. For reliability indices, no consistent threshold values are provided in the psychometric literature. Recommendations vary from 0.60 (Reise et al. 2013) to 0.85 (Stucky and Edelen 2015) for the ECV, and from 0.70 (Reise et al. 2013) to 0.80 (Rodriguez et al. 2016) for Omega Hierarchical. Due to the large number of items (22) inflating the Hierarchical Omega index, we adopted conservative rather than liberal criteria: values ˃ 0.80 for both the Hierarchical Omega and the ECV were considered to be adequate. Such values guarantee simultaneously that data are sufficiently unidimensional, and that the general factor is strong enough and captures a sufficient amount of variance (Reise et al. 2013).
4. For each bifactor model with 22 items, we checked factor loadings of the general factor and tested positive and negative items for systematic differences in their magnitude. As Bonifay (2015) suggests, it is worth inspecting the magnitude of factor loadings of the general factor in the bifactor model. Therefore, if some loadings are systematically lower and these lower loadings are grouped in terms of their content, it might imply that the single general factor was too restrictive and it did not sufficiently explain variance of these items.
5. For each sample, we fitted the two-tier model (Cai 2010, 2016; Bonifay 2015) with two primary dimensions: Self-criticism on which load items of IS, HS, and RS and three specific group factors: IS, HS, and RS (see Fig. 1). To date, the two-tier model (Cai 2010, 2016; Bonifay 2015) has not been used to analyze the structure of the FSCRS. We used GRM estimation. Each two-tier model was compared with the bifactor model by means of the likelihood ratio test.
6. For each sample, we performed the Mokken scale analysis for the 22-item FSCRS, the 14-item Self-criticism subscale, and the 8-item Self-reassurance subscale. Loevinger coefficients of scalability H with standard error, and violations of latent monotonicity were reported. All analyses were performed in the statistical program R 3.1.3, package mokken. For Mokken scales, values of the coefficient H ˃ 0.30 indicate acceptable scalability, values ˃ 0.40 indicate good scalability and values ˃ 0.50 indicate strong scalability (Sijtsma and Molenaar 2002). For an appropriate interpretation of this index, standard errors must be taken into account, because scalability coefficients are ratios, and their standard errors can be large, even for large sample sizes. Therefore, the threshold values above must be corrected according to standard errors to ensure that population values are not different (Kuijpers et al. 2013).

Our general criteria to justify the use of the total score (three subscales together) were as follows: (1) at least acceptable fit of the bifactor model (in terms of all fit indices); (2) the values of the Hierarchical Omega and the ECV indices are above 0.80, (3) the values of the scalability coefficient H for all 22 items scale are above 0.30 taking into account standard errors; and (4) factor loadings of the general factor in the bifactor models are not systematically lower for any sub-dimension. Our general criteria to justify the use of the two general subscores (self-criticism and self-reassurance) were as follows: (1) at least acceptable fit of the two-tier model (in terms of all fit indices); (2) the values of the scalability coefficient H for two sub-dimensions are above 0.30 taking into account standard errors; and (3) the two-tier model has significantly better fit than the bifactor model. All criteria must be satisfied simultaneously.

All the analyses were performed in the thirteen distinct samples from twelve different countries.

## Results

In general, all confirmatory IRT models (except for Canadian two-factor and three-factor models) showed adequate or excellent fit with their respective data (Table 1 shows likelihood-ratio tests directly comparing two-factor, three-factor, bifactor, and two-tier models). All IRT three-factor models had better fit than two-factor models, and all bifactor models had better fit than three-factor models, both in likelihood ratio tests and information criteria (Table 1). In the same way, all two-tier models had better fit than bifactor models, both in likelihood ratio tests and information criteria (Table 1). However, some two-tier models failed to converge because the matrix of their latent dimensions became non-positive definite. This is due to the fact that their primary dimensions were highly correlated. All reliability measures for the total scale and for the subscales were excellent or very good (Tables 2 and 3). Only 4 out of 13 bifactor models failed to satisfy the criteria of simultaneous values of the Hierarchical Omega and the ECV over 0.80: Israel, Netherlands, Slovakia, and United Kingdom 2 (Table 2). However, since the Percentage of Uncontaminated Correlations (PUC) is not very high (0.68), high values of ECV and Hierarchical Omega are important because some amount of correlations is contaminated by correlations among specific factors. Therefore we can conclude that two general factors (self-reassurance and self-criticism with subdimensions IS and HS merged) explain a sufficient amount of variance.

**Table 1:** Fit indices and likelihood-ratio tests of IRT models of 22-item FSCRS

Sample	Model	CFI	TLI	RMSEA	SRMR	AIC	BIC	LogLik	χ^2^ (df)	p
AUS	2-factor	0.95	0.94	0.049	0.064	16 030	15 976	−7 844	–	–
	3-factor	0.95	0.94	0.051	0.057	15 953	15 894	−7 801	86.80 (2)	˂ 0.001
	bifactor	0.98	0.97	0.036	0.051	15 945	15 835	−7 746	108.73 (19)	˂ 0.001
	two-tier	0.96	0.95	0.049	0.064	15 927	15 813	-7 734	24.41 (1)	˂ 0.001
CAN	2-factor	0.90	0.89	0.058	0.065	19 821	19 814	−9 753	–	–
	3-factor	0.88	0.86	0.065	0.059	19 711	19 701	−9 694	118.13 (2)	˂ 0.001
	bifactor	0.93	0.91	0.052	0.055	19 615	19 678	−9 656	75.96 (19)	˂ 0.001
	two-tier	NC	–	–	–	–	–	–	–	–
CH	2-factor	0.97	0.96	0.038	0.086	12 339	12 158	−5 953	–	–
	3-factor	0.95	0.94	0.047	0.058	12 306	12 114	−5 929	48.38 (2)	˂ 0.001
	bifactor	0.96	0.95	0.044	0.041	12 294	12 107	−5 904	50.27 (19)	˂ 0.001
	two-tier	NC	–	–	–	–	–	–	–	–
ISR	2-factor	0.95	0.94	0.049	0.084	22 809	22 850	−11 259	–	–
	3-factor	0.95	0.94	0.046	0.076	22 588	22 628	−11 145	227.93 (2)	˂ 0.001
	bifactor	0.95	0.93	0.053	0.065	22 530	22 557	−11 081	127.54 (19)	˂ 0.001
	two-tier	0.96	0.94	0.048	0.064	22 490	22 517	−11 060	43.08 (1)	˂ 0.001
ITA	2-factor	0.92	0.90	0.054	0.070	20 048	20 037	−9 869	–	–
	3-factor	0.92	0.90	0.054	0.065	19 957	19 955	−9 819	98.65 (2)	˂ 0.001
	bifactor	0.92	0.90	0.056	0.058	19 941	19 912	−9 771	96.53 (19)	˂ 0.001
	two-tier	0.92	0.90	0.058	0.065	19 918	19 887	−9 757	27.92 (1)	˂ 0.001
JAP	2-factor	0.88	0.86	0.056	0.081	14 802	14 683	−7 208	–	–
	3-factor	0.88	0.86	0.057	0.077	14 740	14 614	−7 171	73.58 (2)	˂ 0.001
	bifactor	0.86	0.81	0.065	0.067	14 729	14 585	−7 134	75.33 (19)	˂ 0.001
	two-tier	NC	–	–	–	–	–	–	–	–
NL	2-factor	0.95	0.94	0.042	0.063	18 878	18 859	−9 279	–	–
	3-factor	0.95	0.94	0.042	0.056	18 799	18 777	−9 235	87.90 (2)	˂ 0.001
	bifactor	0.97	0.96	0.034	0.046	18 765	18 708	−9 174	120.92 (19)	˂ 0.001
	two-tier	NC	–	–	–	–	–	–	–	–
POR	2-factor	0.94	0.93	0.057	0.072	36 972	37 096	−18 356	–	–
	3-factor	0.93	0.91	0.063	0.064	36 749	36 875	−18 242	228.28 (2)	˂ 0.001
	bifactor	0.96	0.95	0.048	0.059	36 575	36 712	−18 127	228.42 (19)	˂ 0.001
	two-tier	0.96	0.95	0.047	0.078	36 515	36 653	−18 096	62.70 (1)	˂ 0.001
SVK	2-factor	0.95	0.94	0.041	0.067	74 079	74 635	−36 918	–	–
	3-factor	0.95	0.94	0.044	0.056	73 709	74 274	−36 731	375.31 (2)	˂ 0.001
	bifactor	0.96	0.95	0.041	0.056	73 653	73 889	−36 680	102.13 (19)	˂ 0.001
	two-tier	0.96	0.94	0.043	0.062	73 464	73 701	−36 584	191.77 (1)	˂ 0.001
TAI	2-factor	0.94	0.93	0.048	0.071	20208	20 223	−9 952	–	–
	3-factor	0.94	0.93	0.050	0.066	20 067	20 079	−9 878	148.34 (2)	˂ 0.001
	bifactor	0.96	0.94	0.044	0.053	19 989	19 979	−9 801	154.44 (19)	˂ 0.001
	two-tier	NC	–	–	–	–	–	–	–	–
UK1	2-factor	0.93	0.92	0.054	0.055	82 363	82 589	−41 062	–	–
	3-factor	0.94	0.92	0.053	0.045	81 857	82 445	−40 807	510.71 (2)	˂ 0.001
	bifactor	0.96	0.95	0.045	0.048	81 552	82 235	−40 632	350.20 (19)	˂ 0.001
	two-tier	0.96	0.95	0.054	0.040	81 484	82 006	−40 491	280.53 (1)	˂ 0.001
UK2	2-factor	0.93	0.92	0.053	0.060	46 289	46 436	−23 018	–	–
	3-factor	0.94	0.93	0.049	0.049	45 909	46 057	−22 825	386.18 (2)	˂ 0.001
	bifactor	0.96	0.95	0.044	0.045	45 714	45 879	−22 702	245.77 (19)	˂ 0.001
	two-tier	0.94	0.92	0.052	0.060	45 685	45 850	−22 685	32.94 (1)	˂ 0.001
USA	2-factor	0.95	0.94	0.054	0.084	17 682	17 638	−8 673	–	–
	3-factor	0.93	0.92	0.062	0.070	17 462	17 415	−8 559	228.54 (2)	˂ 0.001
	bifactor	0.95	0.93	0.059	0.070	17 435	17 411	−8 532	54.10 (19)	˂ 0.001
	two-tier	0.94	0.91	0.064	0.080	17 413	17 376	−8 513	37.18 (1)	˂ 0.001

AUS Australia (N = 319); CAN Canada (N = 383); CH Switzerland (N = 230); ISR Israel (N = 476); ITA Italy (N = 389); JAP Japan (N = 264); NL Netherlands (N = 360); POR Portugal (N = 764); SVK Slovakia (*N* = 1326); TAI Taiwan (*N* = 417); UK1 United Kingdom 1 (*N* = 1570); UK2 United Kingdom 2 (*N* = 883) and USA (*N* = 331)

**Table 2:** Reliability measures of 22-items scale FSCRS

Sample	α	ω	ECV	ω_h_
AUS	0.95	0.96	0.90	0.84
CAN	0.92	0.94	0.89	0.81
CH	0.95	0.97	0.92	0.89
ISR	0.90	0.93	0.79	0.71
ITA	0.92	0.94	0.87	0.80
JAP	0.90	0.95	0.88	0.83
NL	0.91	0.93	0.87	0.77
POR	0.92	0.94	0.85	0.80
SVK	0.90	0.94	0.85	0.79
TAI	0.93	0.94	0.91	0.85
UK1	0.94	0.95	0.90	0.84
UK2	0.93	0.94	0.87	0.79
USA	0.93	0.94	0.86	0.80

AUS Australia (N = 319); CAN Canada (*N* = 383); CH Switzerland (*N* = 230); ISR Israel (*N* = 476); ITA Italy (*N* = 389); JAP Japan (*N* = 264); NL Netherlands (*N* = 360); POR Portugal (*N* = 764); SVK Slovakia (*N* = 1326); TAI Taiwan (*N* = 417); UK1 United Kingdom 1 (*N* = 1570); UK2 United Kingdom 2 (*N* = 883) and USA (*N* = 331)

**Table 3:** Reliability measures of subscales of the FSCRS

Sample	IS		RS		HS		IS + HS	
	α	ω	α	ω	α	ω	α	ω
AUS	0.92	0.93	0.90	0.92	0.83	0.88	0.93	0.95
CAN	0.89	0.91	0.86	0.89	0.77	0.84	0.90	0.92
CH	0.90	0.92	0.92	0.94	0.80	0.85	0.92	0.93
ISR	0.89	0.91	0.86	0.89	0.79	0.90	0.89	0.93
ITA	0.90	0.92	0.85	0.88	0.75	0.84	0.91	0.93
JAP	0.81	0.82	0.84	0.86	0.80	0.88	0.88	0.91
NL	0.86	0.87	0.82	0.85	0.80	0.88	0.89	0.91
POR	0.90	0.92	0.88	0.90	0.81	0.90	0.91	0.94
SVK	0.86	0.89	0.83	0.86	0.75	0.82	0.88	0.91
TAI	0.85	0.86	0.88	0.89	0.86	0.89	0.90	0.91
UK1	0.91	0.92	0.88	0.91	0.86	0.91	0.93	0.95
UK2	0.90	0.92	0.85	0.88	0.86	0.91	0.92	0.94
USA	0.90	0.92	0.89	0.90	0.85	0.90	0.92	0.94

AUS Australia (N = 319); CAN Canada (N = 383); CH Switzerland (N = 230); ISR Israel (N = 476); ITA Italy (N = 389); JAP Japan (N = 264); NL Netherlands (N = 360); POR Portugal (N = 764); SVK Slovakia (N = 1326); TAI Taiwan (N = 417); UK1 United Kingdom 1 (N = 1570); UK2 United Kingdom 2 (N = 883) and USA (N = 331)

We also inspected factor loadings of the bifactor model in all 13 samples. In eight out of thirteen samples (except for Canadian, Taiwan, Switzerland, Israeli, and Italian samples), factor loadings of positive items (Self-Reassurance) in the bifactor model were systematically and significantly lower than factor loadings of negative items (IS and HS) suggesting that the single general factor did not sufficiently explain variance of positive items (Table 4). This is another argument for using two general factors.

**Table 4:** Average factor loadings of bifactor models of the FSCRS

	Average factor loadings of bifactor model	
Sample	F (Self-criticism items)	F (Self-reassurance items)
AUS	0.746	0.591*
CAN	0.636	0.595 ns
CH	0.714	0.736 ns
ISR	0.608	0.523 ns
ITA	0.661	0.584 ns
JAP	0.616	0.530 ns
NL	0.746	0.591*
POR	0.715	0.418*
SVK	0.609	0.479*
TAI	0.612	0.640 ns
UK1	0.754	0.560*
UK2	0.709	0.486*
USA	0.680	0.580*

*p < 0.05. AUS Australia (*N* = 319); CAN Canada (*N* = 383); CH Switzerland (*N* = 230); ISR Israel (N = 476); ITA Italy (N = 389); JAP Japan (N = 264); NL Netherlands (N = 360); POR Portugal (N = 764); SVK Slovakia (N = 1326); TAI Taiwan (N = 417); UK1 United Kingdom 1 (N = 1570); UK2 United Kingdom 2 (*N* = 883) and USA (*N* = 331)

After checking the scalability of all FSCRS items by Mokken scale analysis, all FSCRS items are scalable in terms of the H coefficient, but seven scales displayed at least one violation of latent monotonicity (Table 5). Stastistically, this could provide some support for the adequacy of the total score, but it is not decisive. In addition, the authors of the scale (Gilbert et al. 2004) do not recommend using the total score, as it does not make sense from theoretical and clinical points of view. On the other hand, the subscales Self-criticism and Self-reassurance are not only scalable in terms of the H coefficient, but only two of the samples violate the latent monotonicity (Slovak and United Kingdom 1). To conclude, the results show that the use of the overall score cannot be recommended, and in applied research, the use of either two scores (Self-criticism with IS and HS merged, and with RS), or three scores (IS, HS, and RS) is recommended, with the caveat that in nonclinical samples, IS and HS dimensions tend to be very strongly correlated.

**Table 5:** Scalability measures of 22-item FSCRS scale, 14-item Self-criticism, 8-item Self-reassurance

	FSCRS scale (22 items)		Self-criticism (14 items)		Self-reassurance (8 items)	
Sample	coefH (SE)	Monotonicity (# of violations, items)	coefH (SE)	Monotonicity (# of violations, items)	coefH (SE)	Monotonicity (# of violations, items)
AUS	0.495(0.022)	1 (3)	0.556(0.023)	0	0.586(0.026)	0
CAN	0.411(0.020)	0	0.472(0.021)	0	0.483(0.026)	0
CH	0.522(0.025)	0	0.505(0.026)	0	0.648(0.028)	0
ISR	0.343(0.019)	1 (3)	0.448(0.023)	0	0.479(0.025)	0
JAP	0.334(0.026)	0	0.384(0.026)	0	0.424(0.034)	0
ITA	0.399(0.021)	0	0.471(0.023)	0	0.469(0.026)	0
NL	0.355(0.024)	0	0.423(0.027)	0	0.403(0.027)	0
POR	0.391(0.016)	3 (5,18,19)	0.503(0.017)	0	0.528(0.019)	0
SVK	0.325(0.010)	4 (6,9,17,18)	0.396(0.011)	1 (12)	0.414(0.013)	0
TAI	0.401(0.022)	0	0.435(0.022)	0	0.491(0.028)	0
UK1	0.469(0.011)	2 (17,19)	0.544(0.011)	2 (12,17)	0.533(0.012)	0
UK2	0.415(0.015)	1 (17)	0.518(0.015)	0	0.453(0.018)	0
USA	0.417(0.024)	1 (3)	0.513(0.024)	0	0.535(0.026)	0

AUS Australia (N = 319); CAN Canada (N = 383); CH Switzerland (N = 230); ISR Israel (N = 476); ITA Italy (N = 389); JAP Japan (N = 264); NL Netherlands (N = 360); POR Portugal (N = 764); SVK Slovakia (N = 1326); TAI Taiwan (N = 417); UK1 United Kingdom 1 (N = 1570); UK2 United Kingdom 2 (N = 883) and USA (N = 331)

### Data Availability

In order to comply with the ethics approvals of the study protocols, data cannot be made accessible through a public repository. However, data are available upon request for researchers who consent to adhering to the ethical regulations for confidential data.

## Discussion

This study examined the psychometric properties of the FSCRS across 13 different populations and eight language versions using two-factor, three-factor, bifactor, and two-tier models. The main goal was to determine whether the use of two or three separate constructs of Self-criticism (IS and HS) and Self-reassurance were replicated across the populations. An adequate fit was found for bifactor IRT models in all samples, while two-tier models with two primary dimensions demonstrated superior fit in direct comparison with bifactor models. In contrast to those studies supporting a three-factor solution, in which there are two types of self-criticism (IS and HS) and one factor of RS, the results of these analyses suggest a general factor for self-reassurance and one general factor for self-criticism (combining IS and HS). The cross-cultural success of the two-factor model surprised us, as there were more reasons to expect a three-factor model, but the issue remains because measurement model fit is only one consideration among many.

These results are in line with previous studies showing that self-criticism and self-reassurance should be considered as distinct factors (Baião et al. 2015; Longe et al. 2010). They also confirm the distinctivness of these two self-relating processes originally proposed by the authors of the scale (Gilbert et al. 2004). The ability of the FSCRS to assess self-criticism and self-reassurance separately allows both clinicians and researchers to determine whether self-criticism or self-reassurance has shifted due to psychotherapy or experimental manipulations and interventions. In fact, increasing evidence suggests direct effects of the Self-criticism dimension on psychopathology (Baião et al. 2015; Longe et al. 2010) and of the Self-reassurance dimension on well-being (Gilbert et al., 2004, 2017). We therefore recommend using the positive and negative items of the FSCRS as Self-criticism and Self-reassurance separately in both practice and research settings for nonclinical populations. This is because in nonclinical populations hating oneself is relatively rare and therefore leading to floor effects.

However, one caveat is that finding a single self-criticism factor may be the result of a psychometric artefact. Specifically, because all the IS and HS items are negatively worded (contain negative or undesirable content) while all the RS items are positively worded, the FSCRS scale may be unable to differentiate types of self-critcism reliably because respondents are influenced by the larger (perceived) differences between positive and negative items than between types of self-criticism. Reverse-scored, or in this case negative items, very often cluster into a separate factor (Carlson et al. 2011) and these spurious factors are often interpreted substantively while their content co-varies with a reversed or negative item format. This raises the possibility that identification of subscales is methodologically based (Dunbar et al. 2000; Marsh 1996) rather than theoretically. In addition, because the original three-factor solution had acceptable fit, further research on discrimination between self-correcting and self-hating would be desirable especially in relation to psychopathology. Future research should aim to calculate IS and HS separately as well as combined, and examine whether there are differences in the outcomes they predict or respond to specific interventions (e.g., compassion-based interventions).

### Implications

The fact that two general factors for self-criticism and self-reassurance have been confirmed in a large number of diverse samples using a wide range of languages provides preliminary evidence suggesting that this factor structure can be recommended in future research in a range of nonclinical contexts across countries and cultures. Calculating two instead of three scores could be easier and more efficient for both researchers and clinicians. In addition, it is possible that it easier and more helpful to focus on two rather than three factors, but this should be empirically examined. The implications of these findings also extend to the theoretical understanding of self-criticism. IS and HS might not be distinct factors for individuals in nonclinical samples, however, they become distinct in clinical samples because only HS, but not IS, predicts self-harm, depression, anxiety, and stress (Gilbert et al. 2004, 2010; Kupeli et al. 2017; Xavier et al. 2016). Although clinicians sometimes use an overall single score for the FSCRS, Gilbert et al. (2004) does not recommend this and clearly the present results support Gilbert’s view. Our findings suggest that the FSCRS may be useful in determining the etiology of clinical disorders and as an outcome measure of the therapeutic process and therefore based on these findings the use of separate factors of self-criticism and self-reassurance is recommended.

### Future research

Future research is required to further clarify the factor structure of the FSCRS, and particularly to clarify the different structures of this measure in clinical versus nonclinical populations. This is particularly important because different populations might deomstrate different self-critcal processes. In nonclinical samples inadaquacy and inferiority are probably more central and self-hating and wanting to self-harm is not an issue. In future, research should further validate the usefulness of the measure in relation to clinical and other health outcomes (e.g., with physiological measurements such as heart rate variability). Also, future research can also examine self-criticism factors in the context of the original theoretical conceptualizations by Gilbert (2010, 2016) of the evolved basis of self-criticism and self-reassurnace and his proposed tripartite model of affect regulation (threat reward and safeness systems). It has been suggested that self-reassurance is associated with the safeness system, whereas self-criticism is associated with a dynamic interaction between the threat and drive system, where threat plays the dominant role. Future research should examine whether the self-correcting form of self-criticism is also related to the reward system (correcting self in pursuit of reward – e.g., praise, acceptance, achievements), while the hating self form of self-criticism is associated only with the threat system. In addition, future work may need to distinguish much more clearly between self-correction versus the more shame-based self-criticism which is what the scales are designed to focus on (Gilbert 2010).

### Strengths and limitations

All our analyses were performed separately in each sample. We did not merge all samples into one – without testing the invariance of different linguistic versions, such a procedure is not psychometrically valid, and despite its frequent use, it should be avoided (Wendt et al. 2017). Without the invariance testing, we have no evidence concerning the measurement invariance and/or differential test functioning of this instrument across different cultures/languages. Therefore, we have no information concerning possible cross-cultural and/or cross-linguistic biases. Testing the measurement invariance or, ideally, differential test functioning in the IRT context, is beyond the scope of this study and it will be addressed in subsequent research. In fact, Self-criticism and Self-reassurance might have culturally different expressions, so the use of a universal scale to measure these constructs across the world may be inappropriate. However, further research is required to address this issue.

Moreover, as the study includes samples from various countries, varying in size and sampling methods, the conclusions could be threatened due to the differences in the methodologies adopted. The repeatability of the findings across diverse samples and in many different languages reinforces the robust factor structure of the FSCRS, as well as its generalisability. Furthermore, although individual sample sizes were not all large, they all exceeded the minimum number required for sufficient power to run the analyses and the total number, close to 8000, suggests that respondents are likely to be reasonably representative. Nevertheless, as we excluded clinical samples, our findings may not be generalizable to clinical populations.

## Conclusion

The Forms of Self-Criticising/Attacking & Self-Reassuring Scale was found to be a reliable and valid instrument to measure the level of self-criticism and self-reassurance in both the original English language version and in the eight translated versions in nonclinical samples. However, while earlier studies suggest a three-factor solution with two self-criticism subscales (IS and HS), these subscales can also be merged and interpreted as a single general Self-criticism factor, at least in nonclinical samples. Thus, the use of both the three subscales scores and two subscales scores (IS and HS merged) is adequate, although when using the Hated-Self subscale in nonclinical populations researchers should be aware of potential floor effects. Therefore, while for clinical populations we recommend the continued use of three subscales (IS, HS, and RS) based on the previous research, for nonclinical populations we recommend the use of two subscales (Self-reassurance and Self-criticism) based on our findings.
